# ECG Criteria for Left Ventricular Hypertrophy in Hypertensive Black Africans: Insights from the coArtHA Trial

**DOI:** 10.5334/gh.1562

**Published:** 2026-07-21

**Authors:** Annina S. Vischer, Valeriya Nemtsova, Blaise Lukau, Martin Rohacek, Jonathan Macko, Johanna Oehri, Herry Mapesi, Fiona Vanobberghen, Andrew Katende, Ravi Gupta, Herieth Ismael Wilson, Alain Amstutz, Elizabeth Senkoro, Theonestina Byakuzana, Geofrey Mbunda, Jamali Siru, Winfrid Gingo, Niklaus Daniel Labhardt, Maja Weisser, Thilo Burkard

**Affiliations:** 1 Medical Outpatient Department and Hypertension Centre, ESH Hypertension Centre of Excellence, University Hospital Basel, Basel, Switzerland; 2 University of Basel, Basel, Switzerland; 3 SolidarMed, Partnerships for Health, Maseru, Lesotho; 4 Ifakara Health Institute, Ifakara branch, Ifakara, United Republic of Tanzania; 5 Swiss Tropical and Public Health Institute, Allschwil, Switzerland; 6 St. Francis Regional Referral Hospital, Ifakara, United Republic of Tanzania; 7 Division of Clinical Epidemiology, Department of Clinical Research, University Hospital Basel, Basel, Switzerland; 8 Oslo Centre for Biostatistics and Epidemiology, Oslo University Hospital, Oslo, Norway; 9 Bristol Medical School, University of Bristol, Bristol, United Kingdom; 10 Division of Infectious Diseases, University Hospital Basel, Basel, Switzerland; 11 Department of Cardiology, University Hospital Basel, Basel, Switzerland

**Keywords:** hypertension, left ventricular hypertrophy, echocardiography, electrocardiographic criteria, sub-Saharan Africa

## Abstract

**Background:**

Electrocardiographic (ECG) criteria for detecting left ventricular hypertrophy (LVH) have largely been derived from non-African populations. Their diagnostic performance in Black African adults with untreated hypertension remains uncertain.

**Objective:**

To evaluate the diagnostic performance of established ECG-LVH criteria compared with echocardiographically assessed left ventricular mass index (LVMI) in Black African adults with untreated, uncomplicated hypertension enrolled in the coArtHA trial.

**Methods:**

In this subanalysis, we included 1,125 participants from rural Tanzania and Lesotho who underwent baseline 12-lead ECG and focused transthoracic echocardiography (fTTE). LVH was defined as LVMI > 95 g/m² in women, and >115 g/m² in men. Diagnostic performance of multiple ECG-LVH criteria was assessed using correlation analyses, sensitivity and specificity estimates, receiver-operating characteristic (ROC) analysis, including calculation of the area under the ROC curve (AUROC), and precision-recall curves (PRC).

**Results:**

Echocardiographic LVH was present in 56 (5%) participants. Across continuous ECG indices, Cornell voltage product adjusted by +0.8 mV in women showed the strongest association with LVMI (Spearman’s rho = 0.37, *p* < 0.001). Among evaluated criteria, Cornell voltage product-based measures demonstrated the most consistent diagnostic performance across evaluated metrics, with an AUROC of approximately 0.76. Using established cut-off values, this criterion yielded a sensitivity of 64% and a specificity of 80%, whereas voltage-only criteria, including Multi-Ethnic Study of Atherosclerosis (MESA)- and Sokolow–Lyon-based indices, showed limited accuracy in this setting. Exploratory analyses of alternative cut-off values demonstrated the expected sensitivity–specificity trade-offs.

**Conclusions:**

In this cohort of hypertensive Black African adults, ECG criteria showed a moderate association with LVMI by echocardiography. Among established ECG criteria, Cornell voltage product-based measures demonstrated the most consistent diagnostic performance. Diagnostic characteristics were strongly influenced by cut-off selection and clinical context, underscoring the need for population-specific evaluation and external validation of ECG criteria for LVH in African settings.

**Trial registration:**

Clinicaltrials.gov NCT04129840. Registered on October 17, 2019 (https://www.clinicaltrials.gov/).

## Graphical Abstract

**Graphical abstract:**
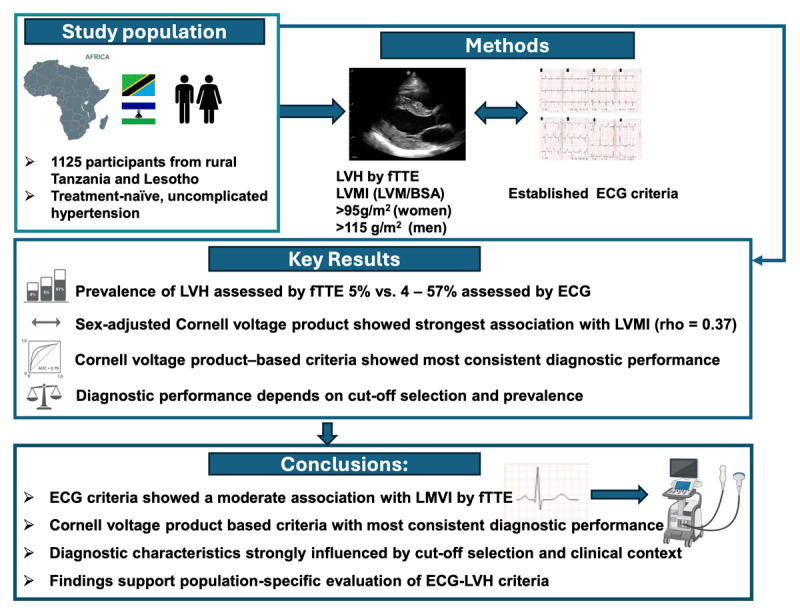
Overview of the study population, methods, and key findings. LVH: left ventricular hypertrophy; fTTE: focused transthoracic echocardiography; LVMI: left ventricular mass index; LVM: left ventricular mass; BSA: body surface area; ECG: electrocardiography.

## Introduction

Left ventricular hypertrophy (LVH) is a recognized complication of hypertension and associated with a worse prognosis, independent of ethnicity (1). Beginning in the middle of the last century, diagnostic options have been investigated to allow non-invasive diagnosis of the presence of LVH and to derive prognostic information. While, initially, electrical activity and remodeling in the electrocardiography (ECG) was compared with anatomical autopsy findings or, as in the case of the Sokolow–Lyon index in 1949, with clinical and radiological findings, more accurate anatomical imaging was subsequently added thanks to the rapid development of transthoracic echocardiography (TTE) and later cardiac magnetic resonance (CMR), resulting in numerous comparisons between the different modalities regarding diagnostic comparability and prognostic value (2,3,4,5,6,7).

While CMR is considered the reference method for assessing left ventricular (LV) mass, its availability in low-resource settings is limited (7). TTE remains the preferred modality in clinical practice, yet access is often restricted in rural regions of sub-Saharan Africa (8,9). In contrast, ECG is inexpensive and more easily available, but its ability to identify LVH varies considerably, and comorbid conditions as well as ethnicity may influence correlation with imaging findings (10,11,12,13,14). Importantly, ECG-based LVH reflects electrical remodeling rather than myocardial mass per se, and complete concordance with imaging-defined LVH is therefore not expected. However, from a daily clinical perspective, one key question is not whether ECG can replicate anatomical imaging but rather which ECG-derived parameter in a given population provides the most useful information for ruling in or ruling out patients with possible hypertensive cardiac involvement in routine care. Most established ECG-LVH criteria were developed and validated in predominantly Caucasian populations, with limited data for African adults and few studies evaluating the suitability of ECG criteria for LVH assessment in Black African populations (3,12,15,16).

The coArtHA trial enrolled treatment-naïve adults with uncomplicated hypertension in Tanzania and Lesotho (17,18). Using this unique dataset, we assessed the diagnostic performance of numerous established ECG-LVH criteria against focused transthoracic echocardiographic (fTTE) left ventricular mass index (LVMI) and explored adapted cut-off values for this population.

## Methods

### Study design and population

The coArtHA trial was an investigator-initiated, open-label, randomized controlled trial conducted in rural Tanzania and Lesotho to compare three antihypertensive treatment strategies (Graphical abstract). Detailed methods and eligibility criteria have been published previously (17,18). In brief, Black African adults (≥18 years) with untreated, uncomplicated hypertension were enrolled. Key exclusion criteria were symptomatic hypertension, recent cardiovascular events, severe comorbidities, pregnancy, or inability to attend follow-up. This subanalysis included all participants with interpretable baseline ECG and fTTE.

All participants gave written informed consent for the collection, storage, and use of clinical data, and sample specimens for research purposes within the coArtHA trial. The study protocol of the coArtHA trial was approved by the Institutional Review Board of the Ifakara Health Institute, the National Health Research Ethics Committee in Tanzania, the Tanzania Medicines and Medical Devices Authority, the National Health Research and Ethics Committee and Ministry of Health of Lesotho, as well as the Ethics Committee of Northwest and Central Switzerland (17,18).

**Figure 1:**
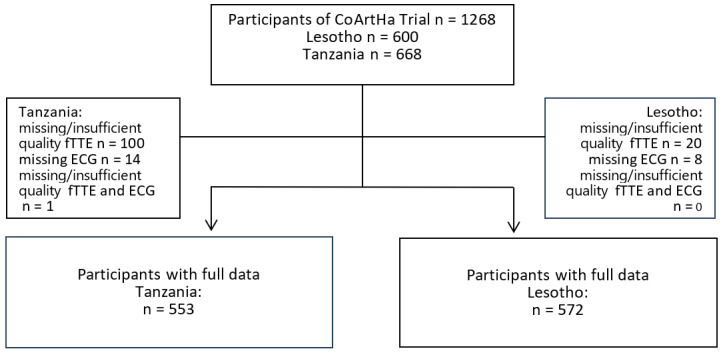
Study flow chart and selection of participants. Legend—fTTE: focused transthoracic echocardiography; ECG: electrocardiography.

This was an exploratory analysis of the coArtHA trial using fTTE and ECG data from baseline investigations. Patients for whom ECG or fTTE data were not available or of insufficient quality were excluded from this analysis (Figure 1).

### Study procedures

#### Blood pressure measurement

Blood pressure (BP) was measured at baseline as standardized, observed office BP following a predefined study standard operating procedure (SOP) according to ESC/ESH 2018 guidelines using an automated device (Omron M6 Comfort, HEM-7321-E) performed by trained study staff (19,20). Details of the BP measurement procedure are provided in the Supplementary Material. Hypertension was defined as systolic ≥140 mmHg and/or diastolic ≥90 mmHg. The average of the last two of three measurements was used for analysis (19).

#### Electrocardiography

A 12-lead ECG was recorded at baseline following an SOP by trained study staff using a Schiller AT-1 electrocardiogram machine at 10 mm/mV calibration and at a speed of 25 mm/s. All ECGs were printed out, including an automated matrix analysis of times and amplitudes, and were verified by trained investigators under cardiologist supervision. Details of the ECG procedure are provided in the Supplementary Material. We compared several established ECG-LVH indices with definitions and thresholds summarized in Table 1. The established ECG-LVH indices were analyzed as both dichotomous and continuous variables to evaluate correlation and discriminative ability versus echocardiographic LVH as appropriate.

**Table 1:** Characteristics of electrocardiographic indexes used in this study.


ECG INDEX	DEFINITION	CUT-OFF VALUE

Cornell voltage criteria (11)	SV_3_ + RaVL	>2.0 mV for women and >2.8 mV for men

Cornell voltage product (19)	(SV_3_ + RaVL)*QRS	>244.0 mVms (19) or >243.6 mVms (56)

Cornell voltage product, adjusted for women 0.6 mV	Men: (SV_3_ + RaVL)*QRS Women: (SV_3_ + RaVL + 0.6 mV)*QRS	>244.0 mVms (8,48) or >243.6 mVms

Cornell voltage product, adjusted for women 0.8 mV	Men: (SV_3_ + RaVL)*QRS Women: (SV_3_ + RaVL + 0.8 mV)*QRS	>244.0 mVms (48) or >243.6 mVms (57)

Novacode 6.1 LVH (58)	RaVL + SV_3_	≥2,600 μV in men ≥2,200 μV in women

RaVL (59,60)		≥1.1 mV

General MESA ECG-LVH (61)	SV_1_ + SV_2_ + RV_5_	≥4.2 mV

Ethnicity- and sex-specific MESA ECG-LVH criterion (11)	SV_1_ + SV_2_ + RV_5_	≥4.8 in men ≥4.3 in women

Sokolow–Lyon voltage (8)	SV_1_ + either RV5/RV6 (whichever is higher)	>3.5 mV

Sokolow–Lyon voltage product (11)	SV_1_ + (RV_5_/RV_6_)*QRS duration	≥371,000 μVms (371 mVms )

Framingham-adjusted Cornell voltage (11,62)	Men: [RaVL + SV_3_ + 0.0174*(age – 49) + 0.191*(BMI – 26.5)]; Women: [RaVL + SV_3_ + 0.0387*(age – 50) + 0.212*(BMI – 24.9)]	Men ≥2.8 mV Women ≥2.0 mV

Minnesota Code 3.1 (63)	RV_5_/V_6_ or RI/II/III/aVF or RaVL	>26 mm >20 mm >12 mm

Minnesota Code 3.3 (63)	RI or R V_5_/V_6_ plus SV_1_; no MC 3.1	>15 mm but ≤20 mm, >35 mm

Strain pattern (52,64)	Coexistence in any of the leads I, II, avL, or V_3_ to V_6_: ST-segment horizontal or downward sloping depression ≥ 0.05 mV, plus negative T-wave; down-sloping convex ST-segment with an inverted asymmetrical T-wave opposite to the QRS axis	Yes/No


RaVL: R wave in aVL; BMI: body mass index; MC: Minnesota Code; ECG: electrocardiography; LVH: left ventricular hypertrophy; MESA: the Multi-Ethnic Study of Atherosclerosis.

#### Focused transthoracic echocardiography

fTTE was performed at baseline following SOP by trained study personnel. In brief, Philips Lumify Ultrasonography devices were used for the fTTE. Regarding LVMI, at least two loops of parasternal long axis views lasting 3 s each were acquired as DICOM files, and exported and synchronized to a cloud drive for data transfer. At University Hospital Basel, DICOM files were analyzed supported by an FDA-approved deep-learning powered software workflow as a decision-support and standardized analysis tool (Us2.ai, Singapore) (21,22). The workflow included automated classification and measurement of the images and videos, followed by mandatory review and approval by an experienced investigator (physician/echotechnician), with corrections applied where necessary, under the supervision of a board-certified cardiologist. The feasibility and reliability of this workflow—combining fTTE by trained study staff with centralized AI-supported analysis and expert approval—have previously been demonstrated in a comparable population-based study conducted in Lesotho (22). Details of the SOP and training are provided in the Supplementary Material.

Left ventricular mass (LVM), LVM indexed by body surface area (BSA) (LVMI), and relative wall thickness (RWT) were calculated according to guideline recommendations: *LVM* = 0.8 × 1.04 × [(*IVS* + *LVID* + *PWT*)^3^ – *LVID*^3^] + 0.6 *grams*, where IVS is interventricular septum diastole (unit mm), LVID is left ventricular end-diastolic diameter (unit mm), and PWT is posterior wall diastole (unit mm); LVMI was defined as LVM/BSA (23). BSA was calculated by the Mosteller formula $\text{BSA}(m^{2})=\sqrt{\frac{\text{height}(\text{cm})\times \text{weight}(\text{kg})}{3600}}$ (24). LVH was defined as LVMI > 95 g/m^2^ in women, and >115 g/m^2^ in men (8).

### Statistics

Normality of continuous variables was assessed using the Shapiro–Wilk test. Continuous data were reported as median (interquartile range, IQR), categorical data as n (% of total). Between-group comparisons were calculated using the Wilcoxon rank-sum test for continuous data and the chi-square test for categorical data. All statistical analyses were conducted using R (version 4.5.2) (25).

#### Testing of ECG parameters

Correlations between ECG parameters and LVMI were calculated with the Spearman method and assessed using Spearman’s rank correlation coefficient (‘ggpubr’ package) (26). Correlation strength was ranked from negligible (0.01–0.19) to very strong (≥0.70) associations (Table S1) (27). Diagnostic performance of ECG criteria for the detection of echocardiographically defined LVH was evaluated by calculating sensitivity, specificity, positive predictive value (PPV), negative predictive value (NPV), and accuracy. Positive and negative likelihood ratios (LR+ and LR–) were calculated as sensitivity/(1 – specificity), and (1 – sensitivity)/specificity, respectively (28).

Receiver-operating characteristic (ROC) curves were plotted, and the area under the ROC curves (AUROC) was calculated using the pROC package (29,30). Discriminative property was graded as follows: an AUROC of 0.5 indicated no discrimination, 0.7 to 0.8 acceptable, 0.8 to 0.9 excellent, and >0.9 outstanding (30). In addition to ROC analysis, we evaluated precision-recall curves (PRC) and the area under the PRC (AUPRC) to account for class imbalance (31,32). Because the baseline AUPRC equals the prevalence LVH, observed AUPRC values were interpreted as enrichment over random classification (31,33). For example, with a prevalence of 5% of LVH, an AUPRC of 0.20 corresponds to a four-fold improvement over chance performance.

#### Selection of ECG parameters and cut-off values

The three ECG parameters with the highest AUROC values were selected for further evaluation of optimized cut-off values within this cohort. In addition, the general Multi-Ethnic Study of Atherosclerosis (MESA) ECG-LVH index, previously shown to perform best in the African American population of the MESA study, was included for comparison (11). For each ECG parameter, diagnostic performance was assessed at the standard guideline-recommended threshold, at thresholds corresponding to 90% and 95% accuracy, 90% and 95% sensitivity, 90% and 95% specificity, and at the cut-off maximizing the Youden index (J), defined as *J* = *sensitivity* + *specificity* – 1. Diagnostic performance of these cut-offs was evaluated using the pROC and PRROC packages (29,34).

#### Assessment of clinical utility

Clinical utility was assessed using decision curve analysis, based on predicted probabilities derived from single-predictor logistic regression models for each ECG parameter (35). Net benefit was evaluated across threshold probabilities ranging from 1% to 20%, with ‘treat all’ and ‘treat none’ strategies used as reference comparators.

#### Internal validation

Internal validation was performed using bootstrap resampling (1,000 iterations) to estimate optimism-corrected discrimination metrics (AUROC and AUPRC) for each ECG criterion (36).

## Results

### Characteristics of the patient cohort

Of the 1,268 coArtHA participants, 1,125 (49% from Tanzania, 51% from Lesotho) had evaluable ECG and echocardiography data (Figure 1). Baseline characteristics were comparable between included and excluded participants, with no relevant differences observed except for country distribution, with a higher proportion of excluded participants from Tanzania (Table S2). Median age was 53.4 (IQR: 44.6–64.4) years, 28% were male, and 38% were HIV positive. LVH by echocardiography was identified in 56 participants (5%), with corresponding within-country prevalences of 8.5% in Tanzania and 1.6% in Lesotho. Participants with LVH were older, more often female, more often with a history of hypertension, and had higher systolic and diastolic BP, and higher BMI compared to those without LVH (Table 2). Differentiation of baseline characteristics between the two countries is shown in Table S3.

**Table 2:** Baseline demographic and clinical characteristics of the study sample, overall and regarding subgroups with echocardiographic signs for LVH.


VARIABLES	OVERALL (n = 1125)	fTTE-LVH PRESENT (n = 56)	fTTE-LVH ABSENT (n = 1069)

Tanzania, n (%)	553 (49.2)	47 (83.9)	506 (47.3)

Lesotho, n (%)	572 (50.8)	9 (16.1)	563 (52.7)

Age, years, median (IQR)	53.4 (44.6–64.4)	59.0 (49.1–68.1)	53.2 (44.3–64.3)

Male, n (%)	314 (27.9)	8 (14.3)	306 (28.6)

HIV positive, n (%)	423 (37.6)	10 (17.9)	413 (38.6)

Previously diagnosed HT, n (%)	469 (41.7)	34 (60.7)	435 (40.7)

Diabetes, n (%)	22 (2.0)	0	22 (2.1)

Diabetes never checked, n (%)	102 (9.1)	3 (5.4)	99 (9.3)

BMI (kg/m^2^), median (IQR)	26.4 (22.8–30.7)	25.1 (21.5–28.1)	26.5 (22.8–30.7)

BSA (m^2^), median (IQR)	1.71 (1.55–1.85)	1.57 (1.47–1.76)	1.70 (1.56–1.85)

Systolic BP (mmHg), median (IQR)	149 (141–163)	165 (152–182)	148 (140–161)

Diastolic BP (mmHg), median (IQR)	99 (93–106)	104 (98–116)	98 (93–105)


Values are presented as n (%), if applicable, with percentages calculated within column groups. fTTE: focused transthoracic echocardiography; HT: hypertension; LVH: left ventricular hypertrophy; BMI: body mass index; BP: blood pressure; BSA: body surface area.

### Prevalence of ECG-LVH markers

The overall prevalence of positive ECG markers according to the presence of echocardiographic signs for LVH is shown in Table 3. The highest prevalence of positive ECG-LVH markers was observed with the general MESA ECG-LVH criterion (633/1125 [56.3%]), the ethnicity- and sex-specific MESA ECG-LVH criterion (566/1125 [50.3%]), and the Sokolow–Lyon voltage index (390/1125 [34.7%]). There were no significant differences in the prevalence of positive values of these criteria, nor for the Framingham-adjusted Cornell voltage index or the Minnesota Code 3.3, between the group of patients with fTTE-LVH and the patients without fTTE-LVH (*p* > 0.05).

**Table 3:** Prevalence of positive ECG markers in the entire cohort, as well as separated for the group with echocardiographic signs of LVH and those without.


INDEX	OVERALL (n = 1125)	ECHOCARDIOGRAPHIC SIGNS FOR LVH (n = 56)	NO ECHOCARDIOGRAPHIC SIGNS FOR LVH (n = 1070)	*p*-value

Cornell voltage criteria, >2.0 mV (women), >2.8 mV (men), n (%)	256 (22.8)	28 (50.0)	228 (21.3)	<0.001

Cornell voltage product ≥ 243.6 mVms, n (%)	103 (9.2)	21 (37.5)	82 (7.7)	<0.001

Cornell voltage product ≥ 244 mVms, n (%)	100 (8.9)	21 (37.5)	79 (7.4)	<0.001

Cornell voltage product ≥ 243.6 mVms, n (%), adjusted 0.6 mV (women)	203 (18.0)	30 (53.6)	173 (16.2)	<0.001

Cornell voltage product ≥ 244 mVms, n (%), adjusted 0.6 mV (women)	197 (17.5)	30 (53.6)	167 (15.6)	<0.001

Cornell voltage product ≥ 243.6 mVms, n (%), adjusted 0.8 mV (women)	255 (22.7)	36 (64.3)	219 (20.5)	<0.001

Cornell voltage product ≥ 244 mVms, n (%), adjusted 0.8 mV (women)	249 (22.1)	34 (60.7)	215 (20.1)	<0.001

Novacode 6.1 LVH, ≥ 2.2 mV (women), ≥ 2.6 mv (men), n (%)	240 (21.3)	30 (53.6)	210 (19.6)	<0.001

RaVL ≥ 1.1 mV, n (%)	190 (16.9)	23 (41.1)	167 (15.6)	<0.001

general MESA ECG-LVH, ≥ 4.2 mV, n (%)	633 (56.3)	33 (58.9)	600 (56.1)	0.784

Ethnicity- and sex-specific MESA ECG-LVH criterion (African American), ≥ 4.3 mV (women), ≥ 4.8 mV (men), n (%)	566 (50.3)	30 (53.6)	536 (50.1)	0.716

Sokolow–Lyon voltage > 3.5 mV, n (%)	390 (34.7)	29 (51.8)	361 (33.8)	0.009

Sokolow–Lyon voltage product, ≥ 371 mVms, n (%)	172 (15.3)	24 (42.9)	148 (13.8)	<0.001

Framingham-adjusted Cornell voltage, ≥ 2.0 mV (women), ≥ 2.8 mV (men), n (%)	564 (50.1)	34 (60.7)	530 (49.6)	0.179

Minnesota Code 3.1, n (%)	270 (24.0)	30 (53.6)	240 (22.5)	<0.001

Minnesota Code 3.3, n (%)	205 (18.2)	4 (7.1)	201 (18.8)	0.419

Strain pattern, n (%)	44 (3.9)	12 (21.4)	32 (3.0)	<0.001


ECG: electrocardiography; LVH: left ventricular hypertrophy; MESA: the Multi-Ethnic Study of Atherosclerosis.

### Characteristics of ECG parameters

Median values of the continuous ECG criteria in the group of participants with fTTE-LVH were significantly higher than in the group without fTTE-LVH, except for the general and male-specific MESA ECG-LVH criterion, and the male- and female-specific Framingham-adjusted Cornell voltage (Table S4).

### Correlations between LVMI and ECG-LVH criteria

Continuous LVH-ECG criteria were compared with continuous LVMI to determine the degree of correlation between these methods and to evaluate the extent to which ECG parameters reflect echocardiographic LV remodeling (Figure S1). Significant, weak to moderate, positive correlations were found between continuous LVMI and all ECG indices, with the highest correlations observed for unadjusted Cornell voltage product, Cornell voltage criteria, 0.6 mV adjusted Cornell voltage product, and R amplitude aVL (rho = 0.373, rho = 0.327, rho = 0.324, and rho = 0.304, respectively; *p* < 0.001 for all). The MESA ECG-LVH criteria showed a significant positive but very weak correlation with LVMI (rho = 0.179, *p* < 0.001).

### Diagnostic performance of guidelines-based ECG-LVH criteria

Sensitivity, specificity, positive (PPV) and negative (NPV) predictive values, and positive (LR+) and negative (LR–) likelihood ratios for LVH were calculated for each ECG criterion with recommended standard cut-off values in comparison to fTTE-LVH (Table 4). The highest LR+ of 7.158 was reached by the strain pattern at a LR– of 0.810, followed by the unadjusted Cornell voltage product at a cut-off of ≥243.6 mVms with an LR+ of 5.074 and LR– of 0.675 (LR+ 4.889 and LR– 0.677 for the cut-off of ≥244 mVms).

**Table 4:** Performance of standard ECG-LVH criteria in overall study population.


PARAMETER	SENSITIVITY (95% CI)	SPECIFICITY (95% CI)	PPV (95% CI)	NPV (95% CI)	ACCURACY	LR+	LR–	TP	FP	TN	FN

Cornell voltage criteria (>2.0 mV women, >2.8 mV men)	0.500 (0.373–0.627)	0.787 (0.761–0.810)	0.109 (0.077–0.154)	0.968 (0.954–0.978)	0.772	2.344	0.636	28	228	841	28

Cornell voltage product (unadjusted) ≥ 243.6 mVms	0.375 (0.260–0.506)	0.923 (0.906–0.938)	0.204 (0.137–0.292)	0.966 (0.953–0.975)	0.896	4.889	0.677	21	82	987	35

Cornell voltage product (unadjusted) ≥ 244 mVms	0.375 (0.260–0.506)	0.926 (0.909–0.940)	0.210 (0.142–0.300)	0.966 (0.953–0.975)	0.899	5.074	0.675	21	79	990	35

Cornell voltage product (women +0.6 mV) ≥ 243.6 mVms	0.536 (0.407–0.660)	0.838 (0.815–0.859)	0.148 (0.106–0.203)	0.972 (0.959–0.981)	0.823	3.31	0.554	30	173	896	26

Cornell voltage product (women +0.6 mV) ≥ 244 mVms	0.536 (0.407–0.660)	0.844 (0.821–0.864)	0.152 (0.109–0.209)	0.972 (0.959–0.981)	0.828	3.429	0.550	30	167	902	26

Cornell voltage product (women +0.8 mV) ≥ 243.6 mVms	0.643 (0.512–0.755)	0.795 (0.770–0.818)	0.141 (0.104–0.189)	0.977 (0.965–0.985)	0.788	3.138	0.449	36	219	850	20

Cornell voltage product (women +0.8 mV) ≥ 244 mVms	0.607 (0.476–0.724)	0.799 (0.774–0.822)	0.137 (0.099–0.185)	0.975 (0.962–0.983)	0.789	3.019	0.492	34	215	854	22

Novacode 6.1 LVH (≥ 2.2 mV women, ≥ 2.6 mV men)	0.536 (0.407–0.660)	0.804 (0.779–0.826)	0.125 (0.089–0.173)	0.971 (0.957–0.980)	0.790	2.727	0.578	30	210	859	26

R amplitude aVL ≥ 1.1 mV	0.411 (0.292–0.541)	0.844 (0.821–0.864)	0.121 (0.082–0.175)	0.965 (0.951–0.975)	0.822	2.629	0.698	23	167	902	33

General MESA ECG-LVH ≥ 4.2 mV	0.589 (0.459–0.708)	0.439 (0.409–0.469)	0.052 (0.037–0.072)	0.953 (0.931–0.969)	0.446	1.050	0.936	33	600	469	23

MESA ECG-LVH African American (≥ 4.3 women, ≥ 4.8 men)	0.536 (0.407–0.660)	0.499 (0.469–0.529)	0.053 (0.037–0.075)	0.953 (0.933–0.968)	0.500	1.068	0.931	30	536	533	26

Sokolow–Lyon voltage > 3.5 mV	0.518 (0.390–0.643)	0.662 (0.633–0.690)	0.074 (0.052–0.105)	0.963 (0.947–0.975)	0.655	1.533	0.728	29	361	708	27

Sokolow–Lyon voltage product ≥ 371 mVms	0.429 (0.308–0.559)	0.862 (0.840–0.881)	0.140 (0.096–0.199)	0.966 (0.953–0.976)	0.840	3.096	0.663	24	148	921	32

Framingham-adjusted Cornell (≥ 2.0 women, ≥ 2.8 men)	0.607 (0.476–0.724)	0.504 (0.474–0.534)	0.060 (0.043–0.083)	0.961 (0.941–0.974)	0.509	1.225	0.779	34	530	539	22

Minnesota Code 3.1	0.536 (0.407–0.660)	0.775 (0.750–0.799)	0.111 (0.079–0.154)	0.970 (0.956–0.979)	0.764	2.386	0.599	30	240	829	26

Minnesota Code 3.3	0.154 (0.062–0.335)	0.758 (0.727–0.785)	0.020 (0.008–0.049)	0.966 (0.949–0.978)	0.739	0.635	1.117	4	201	628	22

Strain pattern	0.214 (0.127–0.338)	0.970 (0.958–0.979)	0.273 (0.163–0.418)	0.959 (0.946–0.970)	0.932	7.158	0.810	12	32	1037	44


NPV: Negative predictive value; PPV: positive predictive value; LR+: positive likelihood ratio (rule-in power); LR–: negative likelihood ratio (rule-out power); TP: true positive; FP: false positive; TN: true negative; FN: false negative.

### Discriminative performance of continuous ECG parameters

**Figure 2:**
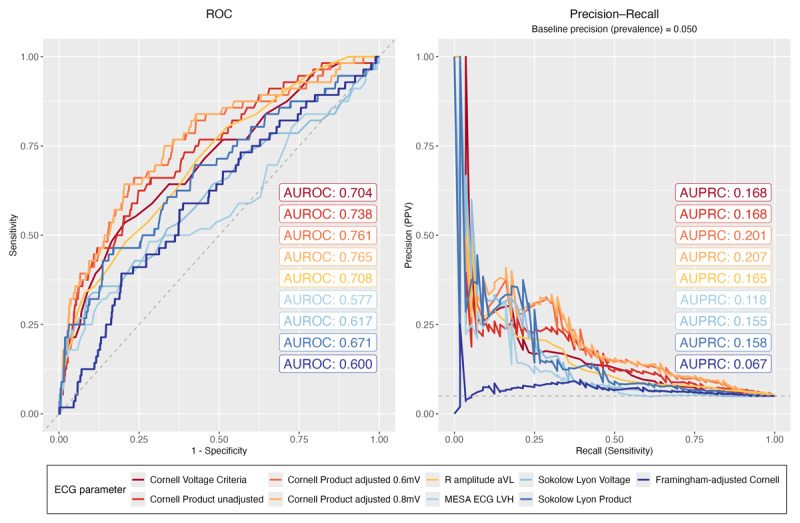
Receiver-operating curves (ROC) with area under the ROC (AUROC) and precision-recall curves (PRC) with area under the PRC (AUPRC) for continuous ECG parameters. Legend—ECG: electrocardiography; LVH: left ventricular hypertrophy; MESA: Multi-Ethnic Study of Atherosclerosis.

Figure 2 shows ROC curves comparing the discriminative ability of continuous ECG parameters for the detection of LVH, providing the basis for potential threshold optimization. Among the indexes investigated, compared to the ‘neutral’ AUROC value of 0.5, the Cornell voltage product adjusted with 0.8 mV and 0.6 mV (women), the unadjusted Cornell voltage product, and the R wave amplitude in aVL had the highest AUROC values (0.765, 0.761, 0.738, and 0.708, respectively) with an acceptable discriminative property (30). The MESA ECG-LVH index showed the lowest AUROC of the tested parameters (0.577).

### Clinical utility testing

**Figure 3:**
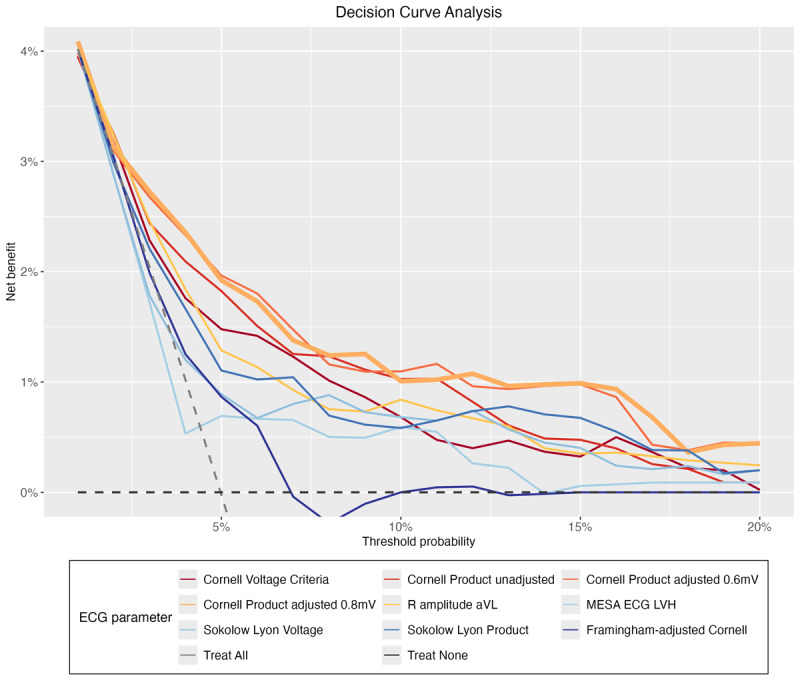
Decision curve analysis of ECG criteria for the detection of LVH. Net benefit is plotted against threshold probability, representing the risk of LVH at which echocardiography would be recommended. Colored curves represent predicted probabilities derived from single-predictor logistic regression models for each ECG criterion. Dashed gray curves indicate reference strategies for treating all patients or treating none. Higher net benefit indicates greater clinical utility.

Across clinically relevant threshold probabilities (approximately 3–10%), Cornell voltage product-based criteria demonstrated consistently higher net benefit in decision curve analysis than voltage-only ECG criteria or alternative ECG markers (Figure 3), particularly within threshold probabilities reflecting low-prevalence settings.

### Internal validation

Bootstrap internal validation showed minimal optimism (<0.01 for all AUROC and AUPRC) for all ECG parameters. Optimism-corrected AUROC and AUPRC values were similar to apparent performance, confirming stable discrimination and minimal overfitting. Cornell voltage product-based criteria—particularly the female-adjusted Cornell product (+0.8 mV)—remained the strongest discriminators after internal validation (Table S5).

### Alternative cut-off values for selected ECG parameters

Based on the previous results, we selected the three best criteria for further exploratory evaluation: Cornell voltage criteria, Cornell voltage product, and R amplitude aVL, in addition to the MESA ECG-LVH index. We analyzed both standard and optimized thresholds for these ECG-LVH criteria to identify the most clinically applicable thresholds (Table S6). The highest LR+ of 19.089 was reached by most tests at their 95% accuracy cut-offs (Cornell voltage product, adjusted +0.6 mV for women, and adjusted +0.8 mV for women, R amplitude aVL (LR– for all 0.949), unadjusted Cornell voltage product and general MESA ECG-LVH (LR– for both 0.983)), and the lowest LR– by the R amplitude aVL at the 95% sensitivity cut-off (0.25 mV, LR– 0.184, LR+ 1.196), the Cornell voltage criteria at the 95% sensitivity cut-off (1.05 mV, LR– 0.219, LR+ 1.152), and the unadjusted Cornell voltage product at the 95% sensitivity cut-off (94.3 mVms, LR– 0.259, LR+ 1.193).

## Discussion

This subanalysis of the coArtHA trial provides several important insights into the diagnostic utility of LVH defined by ECG-LVH compared with LVH defined by fTTE-LVH among hypertensive Black African adults. Overall, the prevalence of fTTE-LVH in this cohort of treatment-naïve adults with uncomplicated hypertension was low (5%), whereas the prevalence of ECG-LVH varied widely across criteria, ranging from 3.9% to 56.6%.

Across all ECG indices, correlations with LVMI were weak to moderate. ECG criteria integrating QRS voltage and duration, particularly Cornell voltage product-based measures and their sex-adjusted versions, showed the strongest and most consistent associations with fTTE-LVH, whereas voltage-only criteria demonstrated limited diagnostic value despite a high prevalence of positive findings. When applying established cut-off values, Cornell voltage product-based criteria provided the most favorable overall discriminative performance and a more favorable balance between potential benefit and misclassification across clinically relevant decision thresholds, particularly in low-prevalence settings.

The limited sensitivity of established ECG-LVH criteria when compared with fTTE-LVH motivated exploratory analyses of alternative threshold definitions. These analyses illustrate the expected sensitivity–specificity trade-offs across different cut-off values and highlight that the diagnostic performance of ECG-LVH criteria relative to fTTE-LVH is strongly influenced by threshold selection, disease prevalence, and clinical context.

The low prevalence of fTTE-LVH observed in this cohort likely reflects the characteristics of the study population. Participants were treatment-naïve adults with uncomplicated hypertension, likely identified early in the disease course through community-based pre-screening, or recruitment of individuals engaged in longitudinal care within HIV or general outpatient clinics (18). In line with this, BP levels were only moderately elevated, and clinically relevant differences in BP were observed between participants with and without fTTE-LVH at baseline. Importantly, the low prevalence of LVH in our study is consistent with recent population-based data from Lesotho, where fTTE-LVH was detected in only a small proportion of adults with hypertensive BP, despite a high burden of other forms of target organ damage such as renal impairment (37). The low prevalence of fTTE-LVH in our cohort influences the interpretation of diagnostic performance metrics, particularly PPV. For this reason, we applied PRC and LR to provide more clinically meaningful insights than AUROC alone, highlighting which ECG criteria truly preferentially identified LVH cases beyond chance performance (28,31,32).

The weak to moderate correlations observed between ECG-LVH indices and fTTE-LVH in the present study are consistent with the underlying biological and methodological differences between these diagnostic measures and with prior reports.

ECG-LVH reflects electrical remodeling of the myocardium, influenced by myocardial conduction properties, wall thickness, and ventricular geometry, whereas fTTE-LVH estimates LVM based on geometric assumptions and image-derived measurements (2,13,38). As shown in previous studies comparing ECG criteria with echocardiography and CMR, concordance between electrical and anatomical markers of LVH is typically modest, and complete agreement is not expected (11,13,14). Importantly, the magnitude of correlation observed in our cohort is comparable to that reported previously from both predominantly non-African populations and African cohorts, using similar methodological approaches (12,14,17,39,40,41,42).

When applying established guideline-based cut-off values, ECG-LVH criteria in the present study showed the typical pattern of limited sensitivity combined with comparatively higher specificity, consistent with previous reports from African populations with hypertension (12,17,40,43,44). In a Nigerian cohort where four ECG criteria were evaluated against echocardiography, both Cornell voltage and Sokolow–Lyon criteria demonstrated lower diagnostic accuracy than observed in our study. However, LVH was defined using a non-sex-specific LVMI threshold (>125/m^2^), which is higher than the currently recommended sex-specific cut-off values applied in the present analysis (16).

In our cohort, ECG criteria incorporating both QRS voltage and duration, particularly Cornell voltage product-based criteria, demonstrated more robust discriminative properties than voltage-only criteria. This finding suggests that accounting for electrical remodeling beyond isolated voltage amplitude may improve the ability of ECG-LVH criteria to reflect structural cardiac involvement, even though overall diagnostic sensitivity remains limited. Findings from other large cohorts support the relative robustness of Cornell-based ECG-LVH criteria across different patient populations (41,44,45,46).

In a subanalysis of African American participants of the LIFE study, both Sokolow–Lyon and Cornell voltage criteria showed ethnic differences in sensitivity and specificity when compared to TTE (47). However, overall discriminative performance assessment by ROC analysis was comparable, with the Cornell voltage product achieving the highest numerical AUROC among evaluated ECG indices. In contrast, ECG-LVH criteria derived from the MESA study, including ethnicity- and sex-specific criteria, demonstrated limited sensitivity and specificity in our cohort, despite favorable performance in African American participants within the original MESA population (11).

This discrepancy may reflect differences in population characteristics, disease stage, and imaging reference standards, as well as the higher prevalence of imaging-defined LVH reported in MESA compared with the present study (11). Together, these observations highlight that ECG-LVH performance is strongly context-dependent and that criteria derived in one population may not be directly transferable to others without external validation. In summary, these findings indicate that while correlations between ECG-LVH indices and fTTE-LVH are modest, and sensitivity at established cut-off values is limited, Cornell-based ECG criteria provide the most robust and consistent signal among established measures, within the constraints imposed by low LVH prevalence.

Despite the distinct pathophysiological basis of ECG-LVH and fTTE-LVH, efforts to refine ECG diagnostic performance through adjusted or population-specific cut-off values are common in the literature, including e.g. sex- and ethnicity-specific thresholds (1,2,10,11,41,47,48). Such approaches aim to improve diagnostic accuracy in settings where access to imaging is limited or where ECG serves as an initial triage tool prior to more advanced imaging (8,9,19,49). Traditionally, ECG-LVH cut-off values have been selected to achieve high specificity, commonly around 90%, reflecting their intended use as confirmatory rather than screening tools (2,42). In the present study, exploratory analyses of alternative thresholds illustrate the expected trade-offs between sensitivity and specificity across different cut-off definitions. These findings emphasize that ECG performance relative to fTTE-LVH is highly dependent on threshold selection and clinical context (e.g. rule-in versus rule-out), rather than supporting a single optimal cut-off applicable across populations.

Importantly, ECG-LVH conveys valuable prognostic information (2,47,50,51,52,53). Studies have demonstrated that ECG-defined LVH is independently associated with adverse cardiovascular outcomes, even when imaging-defined LVH is absent (2,53,54). In the LIFE study, regression of ECG-LVH defined by Cornell voltage criteria, Sokolow–Lyon criteria, or ECG strain pattern was associated with improved cardiovascular outcomes independent of BP reduction (51,52).

These observations underscore that optimizing ECG cut-off values solely to improve agreement with fTTE-LVH may inadvertently attenuate prognostically relevant information inherent to ECG signals. While robust prognostic data from sub-Saharan African cohorts remain scarce, the established prognostic relevance of ECG-LVH in other populations highlights the continued clinical importance of ECG-based assessment in hypertension.

This study has several limitations. First, the prevalence of fTTE-LVH in this cohort of treatment-naïve adults with uncomplicated hypertension was low (5%), resulting in a limited number of participants with LVH. Together with the predominance of female participants, this restricts the generalizability of our findings, particularly to male populations, and underscores the need for validation in additional African cohorts.

Second, the prevalence of echocardiographically defined LVH is dependent on the method of LVM indexation and the applied cut-off values. Studies using different indexation approaches or higher LVMI thresholds have reported substantially different LVH prevalences in African hypertensive populations (14,55). In the present analysis, we applied sex-specific American Society of Echocardiography thresholds, which are widely used in clinical practice and have been linked to adverse cardiovascular outcomes (23). Third, fTTE image acquisition was performed by trained study staff following standardized protocols rather than by board-certified cardiologists. However, all examinations underwent centralized analysis supported by a deep-learning-based decision-support tool, with mandatory review and confirmation by experienced investigators under cardiologist supervision. This workflow has been successfully implemented in previous population-based studies and has been shown to be feasible (22). Finally, the present analysis did not assess the association of ECG-LVH with clinical outcomes such as cardiovascular morbidity or mortality. As a result, the prognostic implications of the identified ECG parameters in sub-Saharan African populations remain to be determined in future longitudinal studies.

In conclusion, in this cohort of treatment-naïve Black African adults with uncomplicated hypertension, ECG-LVH showed a moderate association with fTTE-LVH. Among established ECG-LVH criteria, Cornell-based measures, particularly the Cornell voltage product with adjustment for women, provided the most consistent diagnostic performance when compared with alternative indices. Exploratory analyses indicate that the diagnostic characteristics of ECG-LVH are highly dependent on the selected cut-off values and the intended clinical context. While adjusted thresholds may improve sensitivity or specificity for specific purposes, such adaptations involve trade-offs and may alter the prognostic information conveyed by ECG. These findings highlight the importance of verifying the diagnostic validity of established ECG-LVH criteria in African populations and underscore their potential role in supporting cardiovascular risk assessment in low-resource settings, while acknowledging the need for external validation and outcome-based studies.

## Additional File

The additional file for this article can be found as follows:

10.5334/gh.1562.s1 Supplementary Material. Supplementary methodolical details, Tables S1 – S6 and Figure S1.
