# Supplementary material for: ECG Criteria for Left Ventricular Hypertrophy in Hypertensive Black Africans: Insights from the coArtHA Trial
Source: Glob Heart. 2026 Jul 21;21(1):57. doi: 10.5334/gh.1562 (PMC13398579; doi:10.5334/gh.1562)
Supplement: Supplementary Material. — Supplementary methodolical details, Tables S1 – S6 and Figure S1. [file gh-21-1-1562-s1.pdf]

**Supplementary Material.**

1. Summary of the blood pressure measurement procedure according to the standard operating procedure

Recommendations are based on the Guidelines for the Management of Arterial Hypertension 2018, provided by the European Society of Hypertension (1).

Background:
Correct measurement of blood pressure is a complex task with a 1C Grade evidence for a standardized office measurement and 1A Grade for Out-Of-Office and Self-Monitoring of blood pressure (2). White coat hypertension has shown to be high in an African setting at 15% in a recent meta-analysis (3). On the other hand, 24h-ambulatory blood pressure measurement in a rural population coming from distant areas to the clinic is often not feasible. One randomized study showed comparable differences in blood pressure reduction resulting from office and ambulatory blood pressure measurement (4). For the determination of clinic blood pressure, we will use a standard operating procedure based on the ESC/ESH Guidelines for the management of arterial hypertension 2018 recommendation (1).

Preparation:
Before BP measurement, arm circumference in cm is measured and the size of cuff is determined according recommendation of the BP machine manufacturer (22-42cm: standard cuff, 17-<22cm small cuff). If none is fitting chose the one which is closer to the measured arm circumference. The patient is placed in a comfortable, sitting position with feet on floor, back supported, no caffeine, exercise or smoking in the 30min before measurement, emptied bladder. He/she should wear comfortable clothes, the arm should be supported (e.g. on table or on the thighs). The arm used for measurement should be bare skin, the device directly on the skin (no thin shirts and not just move it up). The patient should not see the screen of the BP machine.

The measurement should be done after 5 minutes in this position only, with a validated blood pressure machine (OMRON M6 comfort). The patient should not talk during the measurement.

At the screening visit, first 2 parallel measurements will be done on each arm to determine the arm with higher values. The reference arm (with the higher blood pressure, if discrepant results between systolic and diastolic measurements, the systolic value will be used to determine the reference arm) will be noted and used for all further clinic BP measurements during the study.

For the determination of baseline office BP three consecutive measurements will be done, spaced 2 min apart and the mean value of the 2nd and 3rd measurement will be calculated. This type of clinic BP has been used in several recent clinical trials and epidemiological studies and is based on the ESH 2018 recommendation (1).

For blood pressure measurement we will use Omron M6 Comfort [HEM-7321-E] using individualized cuff size, which has been validated in similar settings (5).

2. Summary of the electrocardiography procedures according to the standard operating procedure

ECG device and electrodes

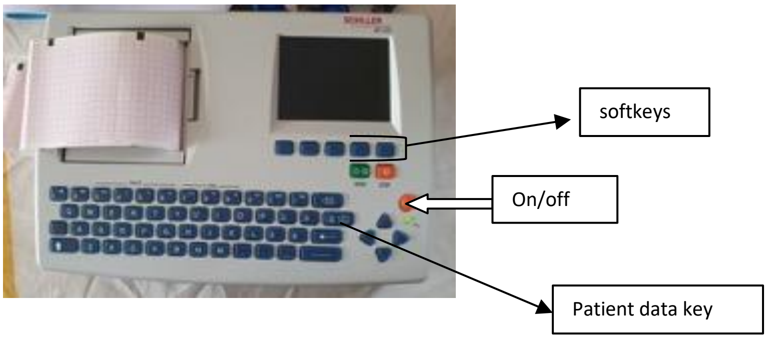


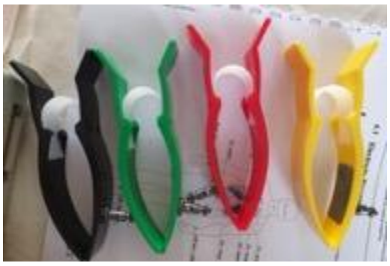

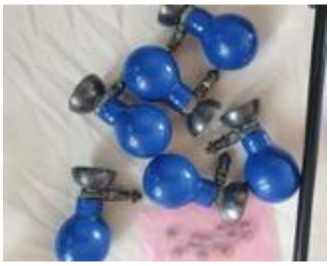

Prepare patient and device

- Explain the procedure to the patient and reassure the patient
- Patient should remove his/her shirt (naked upper body). The patient should lie on his back in a relaxed position on a bed/stretcher.
- Prepare electrode sites (alcohol wipe). If necessary, shave or clip hair on electrode site.
- Add gel on the coloured fixing devices for the extremities and fix them in the correct position (yellow left arm, green left ankle, black right ankle, red right arm).

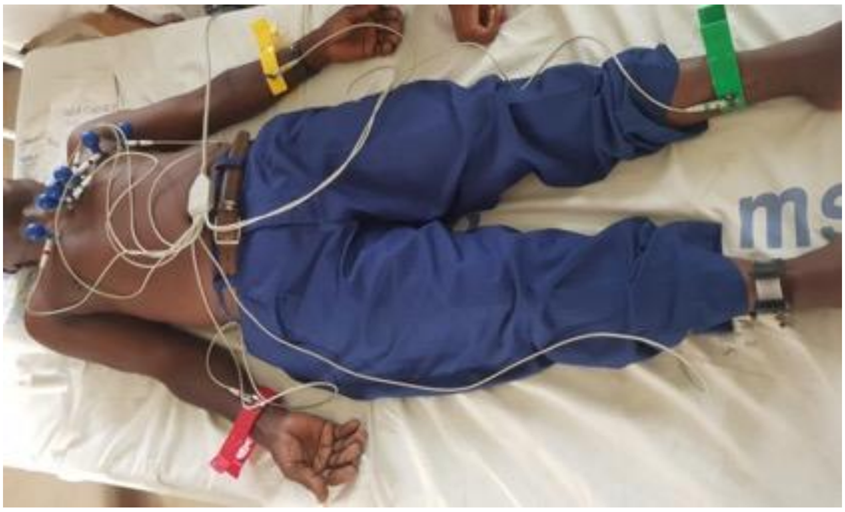

- Before plugging the cables, add the negative-pressure balloons and place them in the correct positions:

1) start with C4 (5th intercostal space (ICS)) in the line of the middle of the clavicle
- Then place:
2) C1 in the 4th ICS parasternal right
3) C2 in the 4th ICS parasternal left
4) C3 between C2 and C4 in equidistance
5) C6 on the patients' side and aligned with C4 (5th ICS axillar line)
6) C5 between C4 and 6 in equidistance

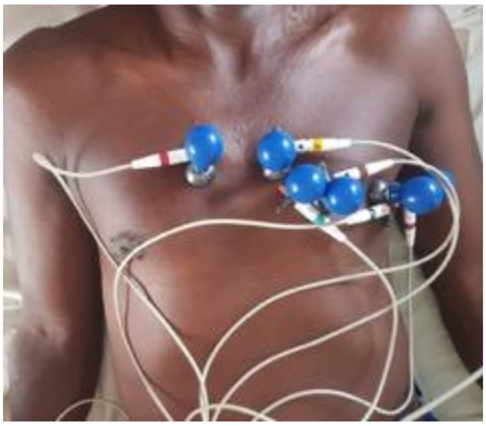

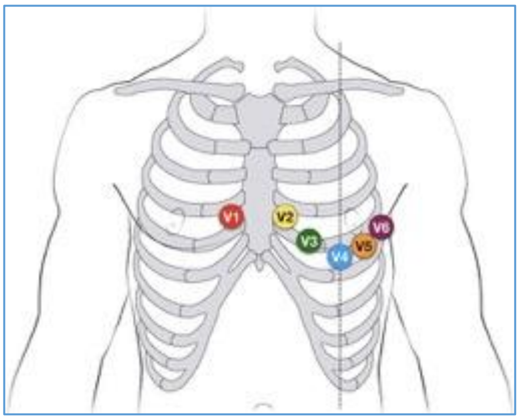


- Plug in the cable to connect with electrodes labelled accordingly with colour codes. Before starting to record enter patient data

<u>ECG Printing</u>

- Before printing, confirm on the screen, if the lead connection is good (clear curves), change the lead group with < and > keys on the key pad, which is available in Nr 1 and 2 on the keypad. You should check lead I, II, III; aVR, aVL, aVf, V1, V2, V3 and V4, V5, V6 for a stable and linear baseline between the QRS complex.
- If the ECG line is irregular or not present on the screen, this indicates, that the electrode resistance is too high or has fallen off and must be repositioned as well.

1. Print out the ECG strip (Start button (green)
2. ECG paper should indicate speed of 25mm/s, amplitude of 10mm/mV, Time and date ECG was taken.

Put the ECG results on the A4 paper, scan it and then put it in the patient's file. On the A4 paper you should indicate the randomization number of the patient, the date and indication if it is the baseline or 24wks ECG. ECG will be fixed on an A4 paper and scanned. The PDF is to be uploaded in the the cloud and a backup is kept in the external HD. Baseline ECGs will be kept in the patient file throughout the study. After obtaining the second ECG at the study end, both ECGs will be stored in a separate folder under the patients' number. ECG will be transferred for analysis to the responsible team under the lead of a board certified cardiologist.

3. Summary of the echocardiography training and procedures according to the standard operating procedure

Study staff training for study procedures including focused transthoracic echocardiography (fTTE) was conducted at the study sites in Ifakara, Tanzania, and Mokhotlong, Lesotho, prior to study initiation. Training was delivered by a physician and experienced cardiac sonographer and followed a standardized curriculum. The training consisted of a five-day on-site program combining theoretical instruction and supervised hands-on practice.

Teaching focused on the correct acquisition of standard views including parasternal long-axis (PLAX) views in the context of the present study using hand-held ultrasound devices. Theoretical teaching covered basic concepts of ultrasound, ultrasound physics, artefacts, transducers, optimization of image quality (e.g. depth and gain), preparation and positioning of the study participant, handling of the tablet and probe, correct probe positioning for PLAX acquisition, identification of relevant cardiac structures, and common pitfalls during image acquisition. Practical hands-on training emphasized supervised acquisition of PLAX views.

Training materials were based on the 2015 Recommendations for Cardiac Chamber Quantification in Adults by the American Society of Echocardiography and the European Association of Cardiovascular Imaging, as well as the Quick Reference Guide from the ASE Workflow and Lab Management Task Force 2018.

Following completion of the training, study staff received the study training materials, including guideline summaries, training presentations, and a standard operating procedure (SOP) to guide image acquisition during the study. The SOP was intended for confidential internal study use only – some figures are blanked in the context of this Supplementary Material.

<u>PLAX acquisition:</u>

All recommendations and measurements are based on the 2015 Recommendations for Cardiac Chamber Quantification in Adults by the American Society of Echocardiography and the European Association of Cardiovascular Imaging, and the Quick Reference Guide from the ASE Workflow and Lab Management Task Force 2018.

Preparation of the patient

Patient lies on lef lateral side, left arm up. Examinator sits on the left side of the patient. Patient takes off the shirt

Preparation of tablet and probe

Switch on tablet, then choose lumify ap

1. Create patient,

2. Press start exam

3. Connect the probe to the tablet

4. Put Jelly on probe

Adjust gain, depth

To acquire loops, press ‘save loop’

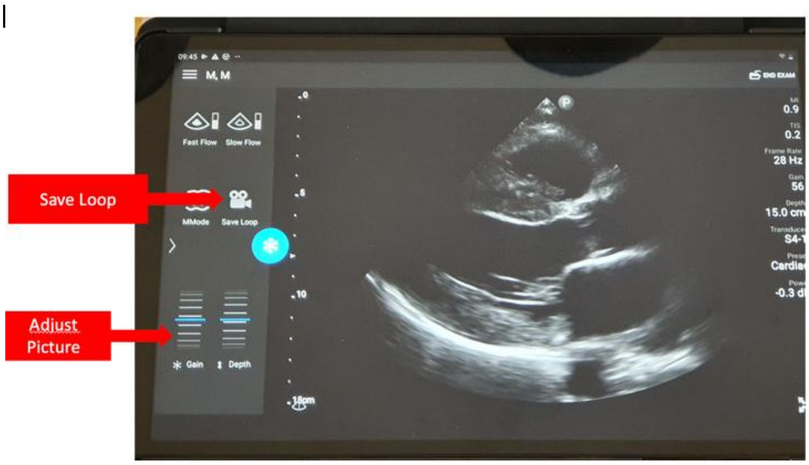


Image Acquisition

Parasternal Long Axis

For acquisition of a parasternal long axis patient should be positioned on the left lateral side. The transducer has to be placed in the 3rd or 4th intercostal space on the left side right close to the sternum and the marker of the transducer (grey stripe on the side) points towards the right shoulder. Depending on the axis of the heart of the subject, the transducer has to be corrected with the aim that the ventricle is as

long stretched as possible focusing on the mid-portion and base of the left ventricle. Usually the apex could not be seen in this view.

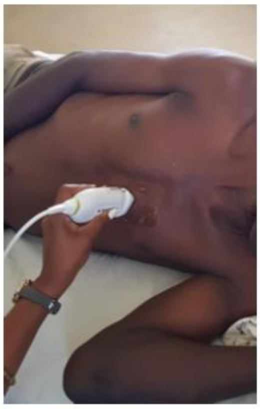
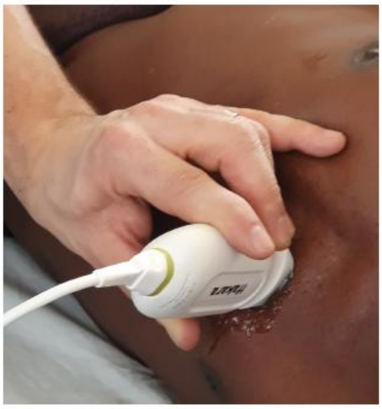
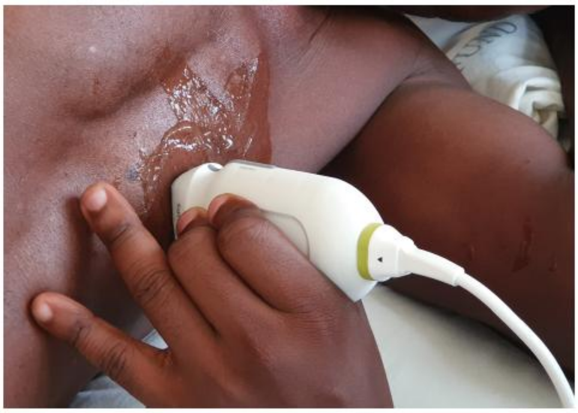

Transducer should be adjusted until the there is no (or minor) angle between the septum and the aortic sinus. The image should be focused on the basal segments of the left ventricle, the aortic valve and the mitral valve together with the left atrium. The aortic valve should close in a symmetric manner with the tips in the middle of the sinus of aortae.

Depth should be adapted, so that one centimeter is at the bottom to visualize structures posterior to the left ventricle.

Linear internal measurements of the LV should be performed in the parasternal long-axis view

2 images illustrating position of the probe in relation to the ultrasound device and how to place measurements according to the guidelines

When the optimal position is found:

- Aquire 2 loops, each with 3 heart cycles (resp. 3 seconds)

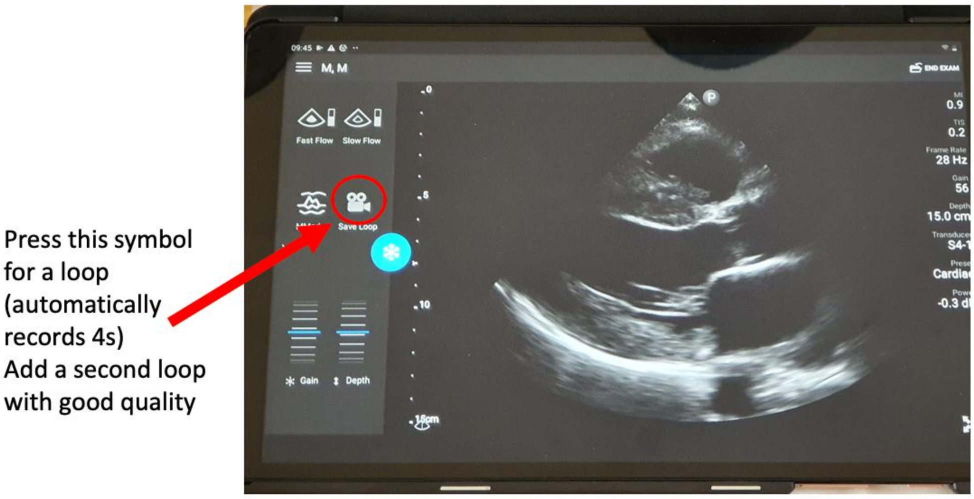


Additional Tables and Figures

Table S1. Interpretation Table of Spearman Rank-Order Correlation Coefficients (6)

| **Spearman ρ** | **Correlation** |
|---|---|
| ≥0.70 | Very strong relationship |
| 0.40-0.69 | Strong relationship |
| 0.30-0.39 | Moderate relationship |
| 0.20-0.29 | Weak relationship |
| 0.01-0.19 | No or negligible relationship |

Note: This descriptor applies to both positive and negative relationships.

Table S2: Baseline demographic and clinical characteristics of the study population, overall and stratified by inclusion status (included vs. excluded due to incomplete ECG or echocardiography data).

| Variables | Overall (n=1268) | Included (n=1125) | Excluded (n=143) |
|---|---|---|---|
| Tanzania, n (%) | 668 (52.7) | 553 (49.2) | 115 (80.4) |
| Lesotho, n (%) | 600 (47.3) | 572 (50.8) | 28 (19.6) |
| Age, years, median (IQR) | 53.6 (44.8 – 64.8) | 53.4 (44.6 – 64.4) | 56.0 (46.3 – 68.6) |
| Male, n (%) | 354 (27.9) | 314 (27.9) | 40 (28.0) |
| HIV-positive, n (%) | 461 (36.4) | 423 (37.6) | 38 (26.6) |
| Previously diagnosed HT, n (%) | 555 (43.8) | 469 (41.7) | 86 (60.1) |
| Diabetes, n (%) | 25 (2.0) | 22 (2.0) | 3 (2.1) |
| Diabetes never checked, n (%) | 116 (9.2) | 102 (9.1) | 14 (9.8) |
| BMI, (kg/$m^2$), median (IQR) | 26.4 (22.8 – 30.7) | 26.4 (22.8 – 30.7) | 26.2 (22.6 – 30.1) |
| BSA, ($m^2$), median (IQR) | 1.69 (1.55– 1.84) | 1.71 (1.55 – 1.85) | 1.70 (1.55 –1.85) |
| Systolic BP (mmHg), median (IQR) | 149 (141 – 163) | 149 (141 – 163) | 152 (143 – 169) |

| | | | |
|---|---|---|---|
| Diastolic BP (mmHg), median (IQR) | 99 (93 –106) | 99 (93 – 106) | 98 (93 –106) |

HT – hypertension, BMI - body mass index, BP- blood pressure, BSA- body surface area

Table S3: Baseline demographic and clinical characteristics of the study sample, overall and stratified for countries

| Variables | Overall (included, n=1125) | Tanzania (n=553) | Lesotho (n=572) |
|---|---|---|---|
| Age, years, median (IQR) | 53.4 (44.6‐64.4) | 55.2 (47.8‐66.2) | 51.7 (41.6‐61.8) |
| Male, n (%) | 314 (27.9) | 145 (26.2) | 169 (29.5) |
| HIV-positive, n (%) | 423 (37.6) | 144 (26.0) | 279 (48.8) |
| Previously diagnosed HT, n (%) | 469 (41.7) | 408 (73.8) | 61 (10.7) |
| Diabetes, n (%) | 22 (2.0) | 17 (3.1) | 5 (0.9) |
| Diabetes never measured, n (%) | 102 (9.1) | 97 (17.5) | 5 (0.9) |
| Height, cm, median (IQR) | 157.0 (151.0‐162.0) | 155.0 (150.0‐160.0) | 159.0 (154.0‐165.0) |
| Weight, kg, median (IQR) | 66.0 (56.0‐77.0) | 63.0 (54.0‐74.0) | 68.0 (58.5‐79.1) |

| | | | |
|---|---|---|---|
| BMI, kg/m², median (IQR) | 26.4 (22.8-30.7) | 26.0 (22.8-30.7) | 26.8 (22.9-30.7) |
| BSA, m², median (IQR) | 1.7 (1.6-1.8) | 1.7 (1.5-1.8) | 1.7 (1.6-1.9) |
| Systolic BP (mmHg), median (IQR) | 149.0 (141.0-163.0) | 154.0 (144.0-169.0) | 146.0 (137.0-156.2) |
| Diastolic BP (mmHg), median (IQR) | 99.0 (93.0-106.0) | 100.0 (92.0-107.0) | 98.0 (93.0-105.0) |
| LVM, g, median (IQR) | 110.7 (94.5-132.0) | 117.3 (98.4-141.4) | 105.9 (90.3-123.9) |
| LVMI, g/m², median (IQR) | 65.3 (56.3-76.9) | 70.9 (61.3-84.7) | 60.7 (52.5-69.3) |
| Relative wall thickness, median (IQR) | 0.4 (0.4-0.5) | 0.4 (0.4-0.5) | 0.4 (0.4-0.5) |

fTTE - focused echocardiography, HT – hypertension, LVH-left ventricular hypertrophy, BMI - body mass index, BP- blood pressure, BSA- body surface area, LVM – left ventricular mass, LVMI – left ventricular mass index.

Table S4: Median values (IQR) for different continuous ECG parameters overall and in participants with and without echocardiographic LVH

| Index | Overall (n=1125) | Echocardiographic signs for LVH (n=56) | No echocardiographic signs for LVH (n=1070) | p |
|---|---|---|---|---|
| Cornell voltage criteria, mV | 1.7 (1.3 – 2.2) | 2.3 (1.7 – 2.8) | 1.7 (1.3 – 2.1) | <0.001 |
| Cornell voltage product, mVms, unadjusted | 142. 8 (103.2 – 188.6) | 199.2 (150.8 – 267.8) | 140.4 (101.4 – 182.4) | <0.001 |
| Cornell voltage product, mVms, adjusted 0.6 mV (women) | 177.6 (140.4 – 223.6) | 246.0 (201.6 – 320.4) | 176.0 (139.4 – 220.8) | <0.001 |
| Cornell voltage product, mVms, adjusted 0.8 mV (women) | 192.4 (150.4 – 240.0) | 206.2 (217.3 – 337.2) | 189.2 (148.2 – 235.6) | <0.001 |
| R amplitude aVL, mV | 0.6 (0.3 – 0.9) | 0.9 (0.6 – 1.4) | 0.6 (0.3 – 0.9) | <0.001 |
| general MESA ECG-LVH, mV | 4.3 (3.5 – 5.4) | 4.8 (3.7 – 6.4) | 4.3 (3.4 – 5.3) | 0.051 |

| | | | | |
|---|---|---|---|---|
| Sokolow-Lyon voltage, mV | 3.1 (2.5 – 3.8) | 3.6 (2.9 – 4.9) | 3.1 (2.5 – 3.8) | 0.003 |
| Sokolow-Lyon voltage product, mVms | 263.2 (207.2 – 328.0) | 318.4 (250.3 – 429.4) | 259.0 (205.2 – 327.6) | <0.001 |
| Framingham-adjusted Cornell voltage, mVms | 2.2 (1.2 – 3.3) | 2.7 (1.7 – 3.8) | 2.2 (1.2 – 3.2) | 0.011 |

ECG- electrocardiography, LVH- left ventricular hypertrophy, MESA- the Multi-Ethnic Study of Atherosclerosis

Table S5: Internal validation using bootstrap resampling with 1000 iterations

| ECG parameter | AUROC (apparent) | AUROC optimism | AUROC corrected | AUPRC (apparent) | AUPRC optimism | AUPRC corrected |
|---|---|---|---|---|---|---|
| Cornell Voltage Criteria | 0.704 | -0.001 | 0.705 | 0.167 | 0.002 | 0.166 |
| Cornell Product unadjusted | 0.738 | 0 | 0.738 | 0.168 | 0.003 | 0.164 |
| Cornell Product adjusted 0.6mV | 0.761 | 0 | 0.761 | 0.201 | 0.004 | 0.197 |
| Cornell Product adjusted 0.8mV | 0.765 | 0 | 0.765 | 0.207 | 0.004 | 0.204 |
| R amplitude aVL | 0.708 | -0.001 | 0.709 | 0.165 | 0.002 | 0.163 |
| MESA ECG LVH | 0.577 | -0.013 | 0.590 | 0.118 | 0.005 | 0.113 |
| Sokolow Lyon Voltage | 0.617 | -0.002 | 0.619 | 0.155 | 0.005 | 0.150 |
| Sokolow Lyon Product | 0.671 | -0.002 | 0.673 | 0.158 | 0.005 | 0.154 |
| Framingham-adjusted Cornell | 0.600 | 0 | 0.599 | 0.067 | 0.004 | 0.063 |

Table S6: Diagnostic performance of selected ECG LVH criteria at different cut-off values.

| Para-meter | Criterion | Cut-off | Specificity (95% CI) | Sensitivity (95% CI) | NPV (95% CI) | PPV (95% CI) | Ac-curacy | LR+ | LR- | TP | FP | TN | FN |
|---|---|---|---|---|---|---|---|---|---|---|---|---|---|
| Cornell voltage criteria | Guideline sex-specific | female >2.0 mV; male >2.8 mV | 0.787 (0.761 – 810) | 0.500 (0.373 – 627) | 0.968 (0.954 – 978) | 0.109 (0.077 – 154) | 0.772 | 2.344 | 0.636 | 28 | 228 | 841 | 28 |
| | Youden | 2.25 | 0.794 (0.769 – 817) | 0.536 (0.407 – 660) | 0.970 (0.957 – 980) | 0.120 (0.085 – 166) | 0.781 | 2.603 | 0.585 | 30 | 220 | 849 | 26 |
| | 90% Accuracy | 2.85 | 0.935 (0.918 – 948) | 0.250 (0.155 – 377) | 0.960 (0.946 – 970) | 0.167 (0.102 – 261) | 0.9 | 3.818 | 0.803 | 14 | 70 | 999 | 42 |
| | 95% Accuracy | Inf | 1.000 (0.996 – 1.000) | 0.000 (0.000 – 064) | 0.950 (0.936 – 961) | NA (NA – NA) | 0.95 | NA | 1 | 0 | 0 | 1069 | 56 |
| | 90% Sensitivity | 1.25 | 0.247 (0.222 – 274) | 0.911 (0.807 – 961) | 0.981 (0.957 – 992) | 0.060 (0.046 – 077) | 0.28 | 1.209 | 0.362 | 51 | 805 | 264 | 5 |
| | 95% Sensitivity | 1.05 | 0.163 (0.142 – 186) | 0.964 (0.879 – 990) | 0.989 (0.960 – 997) | 0.057 (0.044 – 074) | 0.203 | 1.152 | 0.219 | 54 | 895 | 174 | 2 |
| | 90% Specificity | 2.65 | 0.912 (0.894 – 928) | 0.339 (0.229 – 470) | 0.963 (0.950 – 973) | 0.168 (0.110 – 248) | 0.884 | 3.858 | 0.724 | 19 | 94 | 975 | 37 |
| | 95% Specificity | 2.95 | 0.949 (0.934 – 960) | 0.214 (0.127 – 338) | 0.958 (0.945 – 969) | 0.179 (0.106 – 287) | 0.912 | 4.165 | 0.828 | 12 | 55 | 1014 | 44 |

| | | | | | | | | | | | | | |
|---|---|---|---|---|---|---|---|---|---|---|---|---|---|
| Cornell voltage product, unadjusted | Guideline ≥ 244 mVms | 244 | 0.926 (0.909 – 940) | 0.375 (0.260 – 506) | 0.966 (0.953 – 975) | 0.210 (0.142 – 300) | 0.899 | 5.074 | 0.675 | 21 | 79 | 990 | 35 |
| | Guideline ≥ 243.6 mVms | 243.6 | 0.923 (0.906 – 938) | 0.375 (0.260 – 506) | 0.966 (0.953 – 975) | 0.204 (0.137 – 292) | 0.896 | 4.889 | 0.677 | 21 | 82 | 987 | 35 |
| | Youden | 183.2 | 0.752 (0.725 – 777) | 0.625 (0.494 – 740) | 0.975 (0.961 – 983) | 0.117 (0.085 – 158) | 0.746 | 2.521 | 0.499 | 35 | 265 | 804 | 21 |
| | 90% Accuracy | 245.4 | 0.928 (0.911 – 942) | 0.357 (0.245 – 488) | 0.965 (0.952 – 975) | 0.206 (0.138 – 297) | 0.9 | 4.958 | 0.693 | 20 | 77 | 992 | 36 |
| | 95% Accuracy | 426.5 | 0.999 (0.995 – 1.000) | 0.018 (0.003 – 094) | 0.951 (0.937 – 962) | 0.500 (0.095 – 905) | 0.95 | 19.089 | 0.983 | 1 | 1 | 1068 | 55 |
| | 90% Sensitivity | 117.3 | 0.348 (0.320 – 377) | 0.893 (0.785 – 950) | 0.984 (0.966 – 993) | 0.067 (0.051 – 087) | 0.375 | 1.369 | 0.308 | 50 | 697 | 372 | 6 |
| | 95% Sensitivity | 94.3 | 0.207 (0.184 – 232) | 0.946 (0.854 – 982) | 0.987 (0.961 – 995) | 0.059 (0.045 – 076) | 0.244 | 1.193 | 0.259 | 53 | 848 | 221 | 3 |
| | 90% Specificity | 231.2 | 0.901 (0.881 – 917) | 0.411 (0.292 – 541) | 0.967 (0.954 – 976) | 0.178 (0.122 – 253) | 0.876 | 4.142 | 0.654 | 23 | 106 | 963 | 33 |
| | 95% Specificity | 262.8 | 0.950 (0.936 – 962) | 0.304 (0.199 – 433) | 0.963 (0.950 – 973) | 0.243 (0.158 – 355) | 0.918 | 6.123 | 0.733 | 17 | 53 | 1016 | 39 |
| Cornell voltage product, adjusted 0.6 mV (women) | Guideline ≥ 244 mVms | 244 | 0.844 (0.821 – 864) | 0.536 (0.407 – 660) | 0.972 (0.959 – 981) | 0.152 (0.109 – 209) | 0.828 | 3.429 | 0.55 | 30 | 167 | 902 | 26 |
| | Guideline ≥ 243.6 mVms | 243.6 | 0.838 (0.815 – 859) | 0.536 (0.407 – 660) | 0.972 (0.959 – 981) | 0.148 (0.106 – 203) | 0.823 | 3.31 | 0.554 | 30 | 173 | 896 | 26 |
| | Youden | 222.6 | 0.766 (0.740 – 791) | 0.661 (0.530 – 771) | 0.977 (0.965 – 985) | 0.129 (0.095 – 173) | 0.761 | 2.825 | 0.443 | 37 | 250 | 819 | 19 |
| | 90% Accuracy | 281.8 | 0.928 (0.911 – 942) | 0.393 (0.276 – 524) | 0.967 (0.954 – 976) | 0.222 (0.152 – 314) | 0.901 | 5.454 | 0.654 | 22 | 77 | 992 | 34 |
| | 95% Accuracy | 422.3 | 0.997 (0.992 – 999) | 0.054 (0.018 – 146) | 0.953 (0.939 – 964) | 0.500 (0.188 – 812) | 0.95 | 19.089 | 0.949 | 3 | 3 | 1066 | 53 |
| | 90% Sensitivity | 143.4 | 0.285 (0.259 – 313) | 0.893 (0.785 – 950) | 0.981 (0.959 – 991) | 0.061 (0.047 – 080) | 0.316 | 1.249 | 0.376 | 50 | 764 | 305 | 6 |
| | 95% Sensitivity | 121.4 | 0.176 (0.154 – 200) | 0.946 (0.854 – 982) | 0.984 (0.955 – 995) | 0.057 (0.044 – 073) | 0.214 | 1.148 | 0.305 | 53 | 881 | 188 | 3 |
| | 90% Specificity | 263.6 | 0.901 (0.881 – 917) | 0.411 (0.292 – 541) | 0.967 (0.954 – 976) | 0.178 (0.122 – 253) | 0.876 | 4.142 | 0.654 | 23 | 106 | 963 | 33 |
| | 95% Specificity | 296.4 | 0.949 (0.935 – 961) | 0.321 (0.214 – 452) | 0.964 (0.951 – 974) | 0.250 (0.164 – 361) | 0.918 | 6.363 | 0.715 | 18 | 54 | 1015 | 38 |

| | | | | | | | | | | | | | |
|---|---|---|---|---|---|---|---|---|---|---|---|---|---|
| Cornell voltage product, adjusted 0.8 mV (women) | Guideline ≥ 244 mVms | 244 | 0.799 (0.774 – 822) | 0.607 (0.476 – 724) | 0.975 (0.962 – 983) | 0.137 (0.099 – 185) | 0.789 | 3.019 | 0.492 | 34 | 215 | 854 | 22 |
| | Guideline ≥ 243.6 mVms | 243.6 | 0.795 (0.770 – 818) | 0.643 (0.512 – 755) | 0.977 (0.965 – 985) | 0.141 (0.104 – 189) | 0.788 | 3.138 | 0.449 | 36 | 219 | 850 | 20 |
| | Youden | 243.6 | 0.795 (0.770 – 818) | 0.643 (0.512 – 755) | 0.977 (0.965 – 985) | 0.141 (0.104 – 189) | 0.788 | 3.138 | 0.449 | 36 | 219 | 850 | 20 |
| | 90% Accuracy | 297.3 | 0.927 (0.910 – 941) | 0.375 (0.260 – 506) | 0.966 (0.953 – 975) | 0.212 (0.143 – 303) | 0.9 | 5.139 | 0.674 | 21 | 78 | 991 | 35 |
| | 95% Accuracy | 440.1 | 0.997 (0.992 – 999) | 0.054 (0.018 – 146) | 0.953 (0.939 – 964) | 0.500 (0.188 – 812) | 0.95 | 19.089 | 0.949 | 3 | 3 | 1066 | 53 |
| | 90% Sensitivity | 156.4 | 0.304 (0.277 – 332) | 0.893 (0.785 – 950) | 0.982 (0.961 – 992) | 0.063 (0.048 – 082) | 0.333 | 1.283 | 0.352 | 50 | 744 | 325 | 6 |
| | 95% Sensitivity | 121.4 | 0.136 (0.116 – 157) | 0.946 (0.854 – 982) | 0.980 (0.942 – 993) | 0.054 (0.042 – 070) | 0.176 | 1.095 | 0.395 | 53 | 924 | 145 | 3 |
| | 90% Specificity | 277.8 | 0.900 (0.880 – 916) | 0.429 (0.308 – 559) | 0.968 (0.955 – 977) | 0.183 (0.126 – 258) | 0.876 | 4.282 | 0.635 | 24 | 107 | 962 | 32 |
| | 95% Specificity | 310 | 0.950 (0.936 – 962) | 0.339 (0.229 – 470) | 0.965 (0.952 – 974) | 0.264 (0.176 – 376) | 0.92 | 6.843 | 0.695 | 19 | 53 | 1016 | 37 |
| R amplitude aVL | Guideline ≥ 1.1 mV | 1.1 | 0.844 (0.821 – 864) | 0.411 (0.292 – 541) | 0.965 (0.951 – 975) | 0.121 (0.082 – 175) | 0.822 | 2.629 | 0.698 | 23 | 167 | 902 | 33 |
| | Youden | 0.65 | 0.567 (0.537 – 596) | 0.714 (0.585 – 816) | 0.974 (0.959 – 984) | 0.080 (0.059 – 106) | 0.574 | 1.649 | 0.504 | 40 | 463 | 606 | 16 |
| | 90% Accuracy | 1.25 | 0.920 (0.902 – 934) | 0.339 (0.229 – 470) | 0.964 (0.950 – 974) | 0.181 (0.119 – 265) | 0.891 | 4.217 | 0.719 | 19 | 86 | 983 | 37 |
| | 95% Accuracy | 1.95 | 0.997 (0.992 – 999) | 0.054 (0.018 – 146) | 0.953 (0.939 – 964) | 0.500 (0.188 – 812) | 0.95 | 19.089 | 0.949 | 3 | 3 | 1066 | 53 |
| | 90% Sensitivity | 0.35 | 0.284 (0.258 – 312) | 0.911 (0.807 – 961) | 0.984 (0.963 – 993) | 0.062 (0.048 – 081) | 0.316 | 1.273 | 0.314 | 51 | 765 | 304 | 5 |
| | 95% Sensitivity | 0.25 | 0.194 (0.171 – 218) | 0.964 (0.879 – 990) | 0.990 (0.966 – 997) | 0.059 (0.045 – 076) | 0.232 | 1.196 | 0.184 | 54 | 862 | 207 | 2 |
| | 90% Specificity | 1.15 | 0.889 (0.868 – 906) | 0.357 (0.245 – 488) | 0.963 (0.950 – 974) | 0.144 (0.095 – 212) | 0.862 | 3.208 | 0.723 | 20 | 119 | 950 | 36 |
| | 95% Specificity | 1.35 | 0.942 (0.926 – 954) | 0.286 (0.184 – 415) | 0.962 (0.948 – 972) | 0.205 (0.130 – 308) | 0.909 | 4.926 | 0.758 | 16 | 62 | 1007 | 40 |

| | | | | | | | | | | | | | |
|---|---|---|---|---|---|---|---|---|---|---|---|---|---|
| general MESA ECG-LVH | Guideline ≥ 4.2 mV | 4.2 | 0.439 (0.409 – 469) | 0.589 (0.459 – 708) | 0.953 (0.931 – 969) | 0.052 (0.037 – 072) | 0.446 | 1.05 | 0.936 | 33 | 600 | 469 | 23 |
| | Youden | 5.15 | 0.718 (0.691 – 745) | 0.482 (0.357 – 610) | 0.964 (0.948 – 975) | 0.082 (0.057 – 117) | 0.707 | 1.712 | 0.721 | 27 | 301 | 768 | 29 |
| | 90% Accuracy | 6.85 | 0.938 (0.922 – 951) | 0.196 (0.113 – 318) | 0.957 (0.943 – 968) | 0.143 (0.082 – 238) | 0.901 | 3.182 | 0.856 | 11 | 66 | 1003 | 45 |
| | 95% Accuracy | 9.65 | 0.999 (0.995 – 1.000) | 0.018 (0.003 – 094) | 0.951 (0.937 – 962) | 0.500 (0.095 – 905) | 0.95 | 19.089 | 0.983 | 1 | 1 | 1068 | 55 |
| | 90% Sensitivity | 2.35 | 0.059 (0.046 – 075) | 0.911 (0.807 – 961) | 0.926 (0.839 – 968) | 0.048 (0.037 – 063) | 0.101 | 0.968 | 1.515 | 51 | 1006 | 63 | 5 |
| | 95% Sensitivity | 1.25 | 0.007 (0.004 – 015) | 0.964 (0.879 – 990) | 0.800 (0.490 – 943) | 0.048 (0.037 – 063) | 0.055 | 0.972 | 4.772 | 54 | 1061 | 8 | 2 |
| | 90% Specificity | 6.35 | 0.897 (0.877 – 914) | 0.250 (0.155 – 377) | 0.958 (0.944 – 969) | 0.113 (0.068 – 181) | 0.865 | 2.43 | 0.836 | 14 | 110 | 959 | 42 |
| | 95% Specificity | 7.05 | 0.948 (0.933 – 959) | 0.179 (0.100 – 298) | 0.957 (0.943 – 967) | 0.152 (0.084 – 257) | 0.909 | 3.409 | 0.867 | 10 | 56 | 1013 | 46 |

NPV: Negative predictive value; PPV: positive predictive value; LR+: positive likelihood ratio (rule-in power); LR-: negative likelihood ratio (rule-out power), TP: true positive, FP: false positive, TN: true negative, FN: false negative.

Figure S1. Scatter plots showing correlation between continuous ECG parameters and LV mass index from TTE parameters.

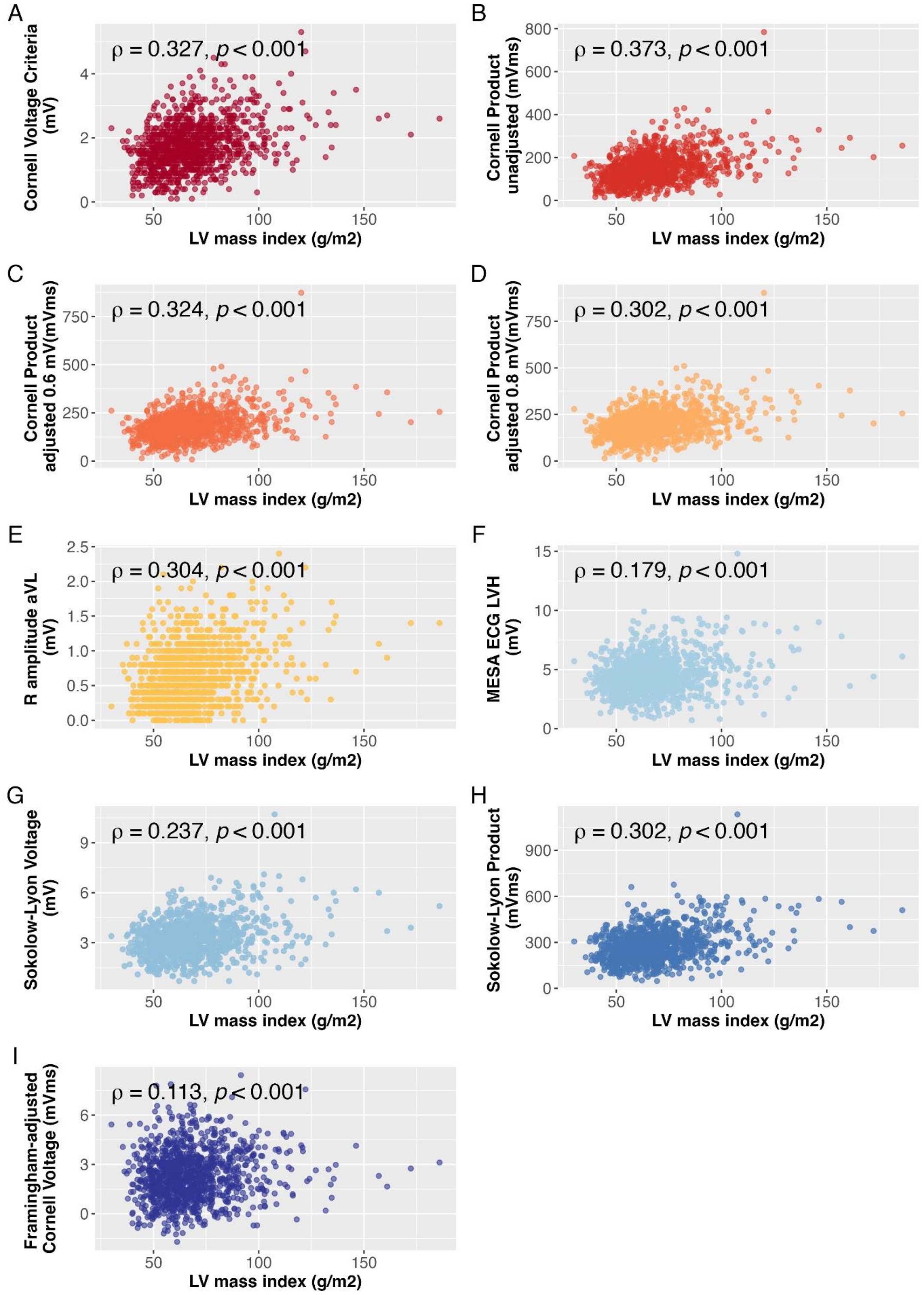


Panel A: Cornell voltage criteria; Panel B: unadjusted Cornell voltage product; Panel C: Cornell voltage product, adjusted for women 0.6 mV; Panel D: Cornell voltage product, adjusted for women 0.8 mV; Panel E: R amplitude in lead aVL; Panel F: General MESA ECG-LVH; Panel G: Sokolow-Lyon voltage; Panel H: Sokolow-Lyon product; Panel I: Framingham-adjusted Cornell voltage

References

1. Williams B, Mancia G, Spiering W, Agabiti Rosei E, Azizi M, Burnier M, et al. 2018 ESC/ESH Guidelines for the management of arterial hypertension. Eur Heart J. 2018;39(33):3021-104.

2. Whelton PK, Carey RM, Aronow WS, Casey DE, Jr., Collins KJ, Dennison Himmelfarb C, et al. 2017 ACC/AHA/AAPA/ABC/ACPM/AGS/APhA/ASH/ASPC/NMA/PCNA Guideline for the Prevention, Detection, Evaluation, and Management of High Blood Pressure in Adults: A Report of the American College of Cardiology/American Heart Association Task Force on Clinical Practice Guidelines. Hypertension. 2018;71(6):e13-e115.

3. Noubiap JJ, Nansseu JR, Nkeck JR, Nyaga UF, Bigna JJ. Prevalence of white coat and masked hypertension in Africa: A systematic review and meta-analysis. J Clin Hypertens (Greenwich). 2018;20(8):1165-72.

4. Ojji DB, Mayosi B, Francis V, Badri M, Cornelius V, Smythe W, et al. Comparison of Dual Therapies for Lowering Blood Pressure in Black Africans. N Engl J Med. 2019;380(25):2429-39.

5. Belghazi J, El Feghali RN, Moussalem T, Rejdych M, Asmar RG. Validation of four automatic devices for self-measurement of blood pressure according to the International Protocol of the European Society of Hypertension. Vasc Health Risk Manag. 2007;3(4):389-400.

6. Leclezio L, Jansen A, Whittemore VH, de Vries PJ. Pilot validation of the tuberous sclerosis-associated neuropsychiatric disorders (TAND) checklist. Pediatr Neurol. 2015;52(1):16-24.
